# Epanchement pleuro-péricardique liquidien révélant un lymphome lymphoblastique

**DOI:** 10.11604/pamj.2014.18.156.2926

**Published:** 2014-06-18

**Authors:** Yves N'da Kouakou N'goran, Kossa Esaïe Soya, Sylvanus Koui Beossin, Ines Angoran, Fatou Traore, Micesse Tano, Yaovi Afassinou, Arnaud Ekou, Florent Koffi, Hermann Yao, Euloge Kouadio Kramoh, Maurice Guikahue Kakou

**Affiliations:** 1 Institut de Cardiologie d'Abidjan, Côte d'Ivoire; 2 Service d'anatomie pathologie CHU de Treichville, Côte d'Ivoire; 3 Service de cardiologie CHU Sylvanus Olympio, Togo

**Keywords:** cœur, lymphome, épanchement pleuro-péricardique, Côte d'ivoire, heart, lymphoma, Pleuro-pericardial effusion, Côte d'ivoire

## Abstract

L'atteinte cardiaque au cours des lymphomes lymphoblastiques est rare. Il s'agissait d'un patient de 17 ans de race noire qui a été admis pour une douleur médiothoracique. Le patient avait des signes d'insuffisance cardiaque droite; un syndrome d’épanchement pleural liquidien gauche; des adénopathies superficielles et une splénomégalie de type IV de Hackett. La biopsie ganglionnaire a été réalisée pour la confirmation du diagnostic par analyse histologique et immuno-histochimique. Des ponctions pleurale et péricardique ont été effectuées. Le patient a été confié au service d'oncologie pédiatrique pour la chimiothérapie. L’évolution a été marquée par le décès du patient 18 jours après. La manifestation cardiaque est une entité rare et très souvent méconnue.la confirmation histologique est parfois difficile à obtenir du vivant de la plupart des patients. Le pronostic est souvent réservé à court terme.

## Introduction

L'atteinte cardiaque au cours des lymphomes lymphoblastiques est rare et se voit souvent à une phase très tardive de l’évolution de la maladie [1]. L'atteinte péricardique est la plus fréquente des situations cardiaques et se traduit très souvent par un épanchement péricardique plus ou moins abondant, responsable parfois de tamponnade et souvent associé à une infiltration tumorale de cette tunique [1, 2]. Nous rapportons un cas d’épanchement pleuro-péricardique liquidien révelant un lymhome lymphoblastique.

## Patient et observation

Il s'agissait d'un patient de 17 ans de race noire qui a été admis pour une douleur médio thoracique. La douleur était de survenue progressive, de type inflammatoire sans irradiation particulière, augmentant d'intensité à l'inspiration profonde et évoluant depuis 02 semaines. A cette douleur était associée une toux sèche, exacerbée par les mouvements, une dyspnée initialement d'effort puis de repos dans un contexte fébrile. Les antécédents étaient sans particularités.

**Figure 1:**
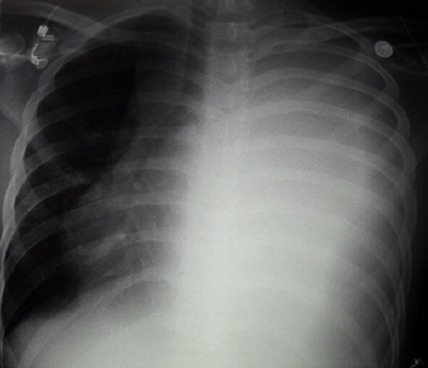
Télécœur de face: opacité dense homogène occupant tout le champ pulmonaire gauche. compression du ventricule droit

**Figure 2:**
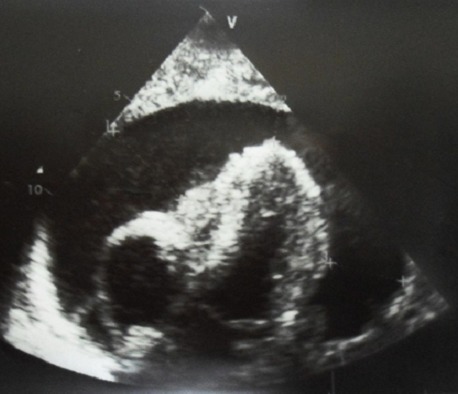
Image échographique: épanchement péricardique de grande abondance avec compression du ventricule droit

A l'examen physique, le patient était dyspnéique au repos avec une pression artérielle à 100/70 mmHg et un pouls à 100bpm. Il y avait des signes d'insuffisance cardiaque droite, Les bruits du cœur étaient assourdis à l'auscultation cardiaque. On notait un syndrome d’épanchement pleural liquidien gauche; des adénopathies, au niveau des aires ganglionnaires superficielles, asymétriques d'environs 3cm de diamètre dures fixées au plan profond et une splénomégalie de type IV de la classification de Hackett. Au télé coeur de face il y avait une opacité dense homogène occupant tout le champ pulmonaire gauche (Figure 1). L’électrocardiogramme objectivait une tachycardie sinusale à 130bpm, un axe QRS normal et un micro voltage diffus. L’écho doppler cardiaque (Figure 2) avait mis en évidence un épanchement péricardique liquidien de grande abondance. L'analyse cytologique et bactériologique des liquides (pleural et péricardique) objectivait un liquide exsudatif à prédominance lymphocytaire avec absence de germe isolé à la culture. Une biopsie ganglionnaire a été réalisée et les examens histologique et immuno-histochimique ont permis de conclure à un lymphome lymphoblastique de phénotype T. A la biologie on avait une anémie hypochrome microcytaire avec un taux d'hémoglobine à 10,9 g/dl et une sérologie au VIH négative. Des ponctions pleurale et péricardique ont été effectuées. Le patient a été confié au service d'oncologie pédiatrique pour la mise en route d'une chimiothérapie. L’évolution a été marquée par le décès du patient dans un contexte d'altération de l’état général 18 jours après.

## Discussion

L'atteinte cardiaque des lymphomes malins non-Hodgkiniens (LNH), bien que rare, est retrouvée fréquemment à l'autopsie des patients décédés de lymphome. Il s'agit le plus souvent de localisations secondaires d'un LNH systémique plutôt que de lymphome cardiaque primitif [3, 4]. Ce dernier se voit essentiellement chez les patients immunodéprimés et sa fréquence est en hausse depuis l'augmentation de la survie des patients porteurs du VIH mais également avec la multiplication des transplantations d'organes et des thérapeutiques immunosuppressives au long cours [4]. La localisation lymphomateuse cardiaque peut se faire soit par dissémination hématogène ou lymphatique, soit par contiguïté à partir d'une tumeur lymphomateuse intrathoracique [5].

Toutes les structures cardiaques peuvent être atteintes avec cependant une nette prédilection pour le péricarde et le myocarde.

La symptomatologie clinique révélatrice de la localisation cardiaque n'est pas spécifique. Elle est en rapport avec la structure cardiaque atteinte [5]. Les signes cliniques sont discrets voire absents dans les LNH cardiaques secondaires, expliquant la discordance entre la fréquence de l'atteinte anatomique (à l'autopsie) et la rareté de la description clinique de ces localisations.

Parmi les manifestations cliniques révélatrices de cette atteinte, on citera: le syndrome d'insuffisance cardiaque congestive en cas d'atteinte myocardique, les troubles du rythme en cas d'atteinte auriculaire ou septale, les signes en rapport avec un épanchement péricardique notamment une tamponnade, un syndrome cave supérieur voire un infarctus du myocarde. Il est cependant intéressant de séparer les LNH cardiaques primitifs, où les manifestations cardiaques sont au premier plan, des localisations secondaires où les manifestations générales ou fonctionnelles du LNH prédominent et où la découverte de l'atteinte cardiaque est souvent fortuite. Dans notre observation l’épanchement pleuro-péricardique était la manifestation principale, associée à une altération de l’état général avec des adénopathies périphériques et une splénomégalie.

Les modifications électrocardiographiques observées ne sont pas spécifiques mais permettent d'attirer l'attention sur une atteinte cardiaque, surtout dans les formes secondaires. Il peut s'agir soit d'un trouble de la conduction [6] ou des signes électriques évoquant un épanchement péricardique (troubles de la repolarisation avec microvoltage) comme dans notre observation. Il est donc important de contrôler régulièrement l’électrocardiogramme chez tout patient porteur de LNH et de réaliser au moindre doute une échographie cardiaque. Cette dernière doit faire partie du bilan préthérapeutique de tout LNH, compte tenu de la toxicité cardiaque potentielle des anthracyclines utilisées dans les protocoles de chimiothérapie [7].

L’échographie cardiaque transthoracique est l'outil de choix pour mettre en évidence l'atteinte cardiaque des LNH qui peut se présenter différemment selon la tunique atteinte. Le plus souvent, elle est sous forme de végétations tumorales fixes ou mobiles, souvent polylobées, siégeant sur l'endocarde valvulaire ou endocavitaire, plus volontiers dans les cavités droites que gauches [8]. L'atteinte péricardique est la plus fréquente et se traduit très souvent par un épanchement péricardique plus ou moins abondant, responsable parfois de tamponnade et souvent associé à une infiltration tumorale de cette tunique [8]. La TDM peut aider au diagnostic en montrant des masses intracardiaques ou péricardiques. Elle fait partie du bilan d'extension du LNH et rend facile le diagnostic étiologique d'une masse cardiaque lorsqu'elle est associée à une atteinte ganglionnaire médiastinale. Mais c'est l'imagerie par résonance magnétique nucléaire (IRM) qui représente le meilleur examen radiologique non invasif pour la détection les localisations tumorales lymphomateuses cardiaques avec notamment une sensibilité de l'ordre de plus de 90% [1, 2]. Dans le cas clinique décrit, nous n'avons pas réalisé de scanner thoracique ni d'IRM mais plutôt une biopsie ganglionnaire a été réalisée et les examens histologique et immuno-histochimique ont permis de conclure à un lymphome lymphoblastique de phénotype T. L'immunohistochimie permet actuellement une confirmation rapide du diagnostic de lymphome. L'aspect histologique retrouvé est le plus souvent celui d'un lymphome B à grandes cellules, de haut grade de malignité ou de malignité intermédiaire. Ceux du sujet immunodéprimé sont souvent de type immunoblastique [1].

Le pronostic des atteintes cardiaques lymphomateuses reste très réservé même si des rémissions prolongées sont parfois rapportées [1, 9, 10]. En effet, la grande majorité des observations rapportées dans la littérature font part du décès précoce des patients, le retard diagnostique en est sûrement la cause principale.

Le traitement des localisations cardiaques du LNH fait appel à la chimiothérapie habituelle des lymphomes, associée ou non à la radiothérapie. Les résultats sont cependant moins encourageants, comparés à ceux des autres formes systémiques des lymphomes [8].

## Conclusion

La manifestation cardiaque est une entité rare et très souvent méconnue. La confirmation histologique est parfois difficile à obtenir du vivant de la plupart des patients. Le pronostic est souvent réservé à court terme car le diagnostic est très souvent tardif et la réponse à la chimiothérapie rarement complète et durable. La nécessité d'y penser pour un diagnostic précoce et une prise en charge précoce et efficace est donc nécessaire.
